# Myeloid cells protect corneal nerves against sterile injury through negative-feedback regulation of TLR2–IL-6 axis

**DOI:** 10.1186/s12974-023-02710-3

**Published:** 2023-02-07

**Authors:** Hyun Ju Lee, Hyeon Ji Kim, Jung Hwa Ko, Joo Youn Oh

**Affiliations:** 1 Laboratory of Ocular Regenerative Medicine and Immunology, Biomedical Research Institute, Seoul National University Hospital, 101 Daehak-Ro, Jongno-Gu, Seoul, 03080 South Korea; 2 Department of Ophthalmology, Seoul National University College of Medicine, 103 Daehak-Ro, Jongno-Gu, Seoul, 03080 South Korea

**Keywords:** Cornea, Interleukin 6, Myeloid cell, Nerve, Neutrophil, Toll-like receptor 2

## Abstract

**Background:**

Mounting evidence suggests that the immune system plays detrimental or protective roles in nerve injury and repair.

**Main body:**

Herein we report that both CD11b^hi^Ly6G^hi^ and CD11b^hi^Ly6C^hi^Ly6G^lo^ myeloid cells are required to protect corneal nerves against sterile corneal injury. Selective depletion of CD11b^hi^Ly6G^hi^ or CD11b^hi^Ly6C^hi^Ly6G^lo^ cells resulted in aggravation of corneal nerve loss, which correlated with IL-6 upregulation. IL-6 neutralization preserved corneal nerves while reducing myeloid cell recruitment. IL-6 replenishment exacerbated corneal nerve damage while recruiting more myeloid cells. In mice lacking Toll-like receptor 2 (TLR2), the levels of IL-6 and myeloid cells were decreased and corneal nerve loss attenuated, as compared to wild-type and TLR4 knockout mice. Corneal stromal fibroblasts expressed TLR2 and produced IL-6 in response to TLR2 stimulation.

**Conclusion:**

Collectively, our data suggest that CD11b^hi^Ly6G^hi^ and CD11b^hi^Ly6C^hi^Ly6G^lo^ myeloid cells confer corneal nerve protection under sterile injury by creating a negative-feedback loop to suppress the upstream TLR2–IL-6 axis that drives corneal nerve loss.

## Background

The innate immune system is the first line of host defense in response to tissue perturbation (e.g., infection, injury); it participates in both inflammatory and homeostatic processes that eliminate the source of perturbation and enforce the functional and structural integrity of tissues [1]. In the course of injury to the nervous system, innate immune cells, including myeloid-derived cells (e.g., neutrophils, monocytes/macrophages) and tissue-endogenous cells (e.g., resident macrophages, microglia), rapidly infiltrate and are activated at sites of injury where they exert either deleterious or beneficial effects on nerves, depending on the context [2–21]. Most studies thus far have focused on elucidating the functions of innate immune cells in neurodegeneration and remyelination in diseases of the central nervous system (CNS). The roles of innate immunity in the injured peripheral nervous system (PNS), however, are less well-established.

The cornea is one of the most densely innervated tissues in the body and is richly supplied by sensory nerve fibers that form part of the PNS [22–24]. Resident and recruited immune cells on the ocular surface play a key role in the maintenance of ocular homeostasis (reflected by corneal transparency) or in the induction of vision-compromising diseases (represented by corneal opacification) [25, 26]. In fact, abnormal immune responses are considered to underlie most ocular surface disorders including both inflammatory and degenerative corneal diseases. At the same time, corneal innervation is often lost or compromised in patients with a variety of ocular surface disorders, and corneal nerve dysfunction is a frequent feature that causes corneal opacity, one of the major causes of visual impairment worldwide [22–24, 27]. Together, these findings suggest that the inflammatory and neurodegenerative pathways in the cornea might be intertwined. An outstanding question therefore is whether and how the immune system affects corneal nerves in the course of disease.

The goals of this study were to investigate the effects of innate immune cells (myeloid cells marked by CD11b, Ly6G or Ly6C positivity) on corneal nerves in a mouse model of sterile corneal injury (chemical injury followed by mechanical injury) and to explore the upstream pathway that drives corneal nerve loss and regulates myeloid cell recruitment under injury.

## Materials and methods

### Animals and animal model

All of the animal procedures were approved by the Institutional Animal Care and Use Committee of Seoul National University Hospital Biomedical Research Institute (Seoul, Korea) (IACUC Nos. 19-0069, 20-0089, 21-0275) and were performed in strict accordance with the ARVO (Association for Research in Vision and Ophthalmology) statement on the use of animals in ophthalmic and vision research.

BALB/c and C57BL/6 mice were purchased from KOATECH (Pyeongtaek, Korea). Toll-like receptor 2 (TLR2) knockout (KO) mice and TLR4 KO mice (C57BL/6 background) were obtained from the Jackson Laboratory (Bar Harbor, ME). The mice were maintained in a specific pathogen-free environment and freely fed a normal diet of laboratory rodent chow (38,057, Cargill Agri Purina, Seongnam, Korea) and water. Eight-week-old male mice were used in all of the experiments.

After anesthesia by intramuscular injection of zolazepam–tiletamine (Zoletil^®^, Virbac, Carros, France) and topical instillation of 0.5% proparacaine hydrochloride ophthalmic solution (Hanmi Pharm., Seoul, Korea), injury was made to the right eye by applying absolute ethanol to the whole cornea for 15 s followed by thorough irrigation with 2 mL Hank’s balanced salt solution under a surgical operating microscope. Then the whole corneal epithelium including the limbal epithelium was scraped using a #11 surgical blade.

For CD11b^hi^Ly6G^hi^ or CD11b^hi^Ly6C^hi^ cell depletion, anti-mouse Ly6G mAb (100 μg/100 μL, clone 1A8, BioXCell, West Lebanon, NH), anti-mouse Ly6C mAb (200 μg/100 μL, clone Monts 1, BioXCell) or the same amount of rat IgG2a isotype control (clone 2A3, BioXCell) was administered by intraperitoneal (IP) injection at day 0 (immediately after injury), day 2 and day 4.

For IL-6 neutralization, either anti-mouse IL-6 Ab (5 μg/5 μL, R&D Systems, Minneapolis, MN) or isotype control IgG (R&D Systems) was subconjunctivally administered using a 31-gauge Hamilton syringe at day 0 (immediately after injury), day 2 and day 4 under an operating microscope.

For IL-6 addition, recombinant mouse IL-6 (100 ng in 5 μL phosphate-buffered solution (PBS), BioLegend, San Diego, CA) or the same volume of PBS was injected into the subconjunctival space using a 31-gauge Hamilton syringe immediately after injury.

### Clinical ocular examination

The eyes were serially observed under slit-lamp biomicroscopy and photographed with a camera mounted on a surgical microscope.

Corneal opacity was clinically graded based on the standardized opacification scoring system (grade 0: no opacity; grade 1: mild opacity, iris details visible; grade 2: moderate opacity, pupillary margin visible, iris details invisible; grade 3: significant opacity, pupillary margin invisible; and grade 4: completely opaque, pupil and anterior chamber not visible) [28].

Corneal epithelial defect was observed after corneal vital staining with 3% (v/v) lissamine green dye or 0.25% (v/v) fluorescein dye, and the extent of staining was graded using the standardized scale system (score 0: no staining; score 1: less than one-third; score 2: less than two-thirds; and score 3: more than two-thirds staining of cornea) [29].

The extent of corneal neovascularization was clinically scored on a scale of 0–3 for each quadrant using the standardized scale system (scale 0: no new vessels; scale 1: new vessels at corneal limbus; scale 2: new vessels spanning limbus and approaching corneal center; scale 3: new vessels spanning center). Then, the scores for each quadrant were summed to yield the overall corneal neovascularization score (range: 0–12) for each eye [30].

### Immunostaining of corneal whole-mounts

The corneas were excised, fixed in acetone/methanol (1:1) at room temperature (RT) for 10 min, and rinsed in PBS. After 2% bovine serum albumin blocking at RT for 1 h, the tissues were incubated at 4 °C overnight with anti-β III tubulin Ab (1:200, MilliporeSigma, Burlington, MA), anti-Ly6G Ab (1:200, BD Horizon, San Jose, CA), anti-Ly6C Ab (1:200, BD Horizon) or anti-IL-6 Ab (1:100, Bioss Antibodies Inc., Woburn, MA). After PBS washing, the tissues were transferred onto slides, incised into 4 quadrants and flat-mounted with Dako Faramount Aqueous Mounting Medium (Agilent, Santa Clara, CA). The stained corneal whole-mounts were examined and photographed under fluorescence confocal microscopy (BX53-DP74, Olympus, Tokyo, Japan). The microscope and software settings were identical for all of the samples within the experiments.

All of the images were analyzed independently by two masked researchers (H.J.L. and J.Y.O.) using ImageJ software (NIH, Bethesda, MD, USA). After background subtraction and thresholding, the stained area was quantified by calculation of the percentage threshold area positive for β III tubulin, Ly6G or Ly6C on the acquired whole-mount images (defined as the percentage of β III tubulin, Ly6G or Ly6C pixels divided by the total number of pixels per field of view).

### Keratocyte culture and TLR2 stimulation

Primary keratocytes isolated from human cornea were purchased from ScienCell Research Laboratories (Carlsbad, CA) and cultured in Fibroblast Medium (ScienCell Research Laboratories). For TLR2 stimulation, the cells were treated with 1–100 ng/mL Pam2CSK4 (Invivogen, San Diego, CA) for 24 h and were subjected to assays.

### Flow cytometry

The peripheral blood was collected, treated with RBC lysis buffer for 5 min, and centrifuged at 2000 rpm for 10 min. The resultant blood cells were stained with Abs against CD11b, Ly6G or Ly6C (eBioscience, San Diego, CA) at 4 °C for 30 min. Keratocytes were stained with anti-TLR2 Ab, anti-TLR3 Ab and anti-TLR4 Ab (Invitrogen, Waltham, MA). The stained cells were assessed for fluorescence using a S1000EXi Flow Cytometer (Stratedigm, San Jose, CA). The data were analyzed using the FlowJo program (Tree Star, Ashland, OR).

### Real-time RT-PCR

The corneas were cut into small pieces by microscissors, incubated in RNA isolation reagent (RNA Bee, Tel-Test Inc., Friendswood, TX) and homogenized with an ultrasound sonicator (Ultrasonic Processor, Cole Parmer Instruments, Vernon Hills, IL). Total RNA was extracted using the RNeasy Mini kit (Qiagen, Valencia, CA), and first-strand cDNA was synthesized by reverse transcription (High Capacity RNA-to-cDNA™ Kit, Applied Biosystems, Carlsbad, CA). Real-time amplification was performed in TaqMan^®^ Universal PCR Master Mix (Applied Biosystems) in an automated instrument (ABI 7500 Real Time PCR System, Applied Biosystems). All of the PCR probe sets were purchased from Applied Biosystems (TaqMan^®^ Gene Expression Assay kits). The data were normalized to GAPDH and expressed as fold changes relative to controls.

### ELISA

Cell-free supernatants were acquired from keratocyte cultures by centrifugation and measured for IL-6 level using DuoSet^®^ ELISA kits (R&D Systems).

### Statistical analysis

Statistical tests and generation of graphs were performed using Prism software (GraphPad, San Diego, CA). After the D’Agostino and Pearson test or Shapiro–Wilk test for normal distribution of data, one-way ANOVA with Tukey’s test or Kruskal–Wallis test with Dunn’s multiple-comparisons test was applied for comparison of mean values from more than two groups. Mann–Whitney U test was used for comparison of the means of two groups. All of the data are presented as mean ± SD. Differences were considered significant at *p* < 0.05.

## Results

### Time courses of corneal nerves and myeloid cells after injury

**Fig. 1:**
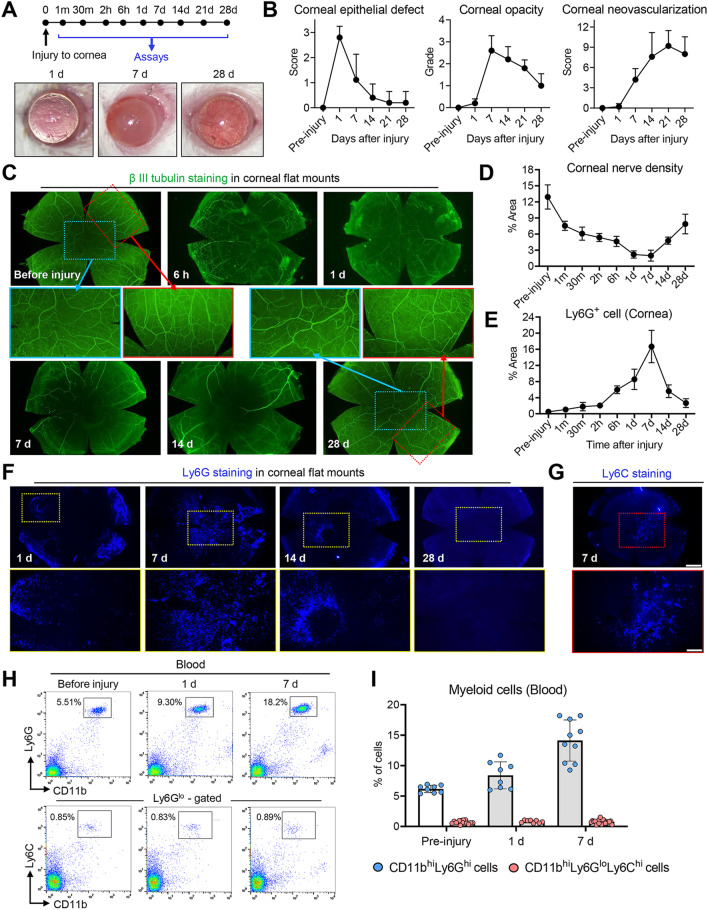
Time courses of corneal disease manifestations, corneal nerve density, and cornea-infiltrating and circulating myeloid cells after sterile injury to cornea. **A** Experimental time frame and representative corneal photographs at 1, 7 and 28 d post-injury. **B** Time courses of corneal epithelial defect, stromal opacity and neovascularization as graded by standardized scoring systems. *n* = 5 at each time-point. **C**, **D** Representative β-tubulin III staining images of corneal whole-mounts at indicated time-points post-injury (**C**). Amplified views of corneal center (blue box) and of corneal quadrant oriented to limbal margins at top (red box). Quantification of corneal nerve density expressed as percentage of threshold area positive for β-tubulin III signal per field of view (**D**). *n* = 8 at each time-point. **E**, **F** Representative images of Ly6G staining of corneal whole-mounts with each amplified view (yellow box) (**F**). Quantification of Ly6G^+^ cell infiltration into cornea measured as percentage of threshold area positive for Ly6G signal per field of view (**E**). *n* = 8 at each time-point. **G.** Representative image of Ly6C-stained corneal whole-mounts with amplified view of corneal center (red box) at 7 d post-injury. **H**, **I** Representative and quantitative flow cytometry results for CD11b^hi^Ly6G^hi^ cells and CD11b^hi^Ly6C^hi^Ly6G^lo^ cells in peripheral blood before injury and at 1 and 7 d post-injury. Each circle represents the data from an individual animal. Mean values + SD are shown in **B**. Mean values ± SD are shown in **D**, **E** and **I**

To assess the loss of corneal nerves and the influx of myeloid cells following injury, we first defined the time courses of corneal nerve density, cornea-infiltrating and blood-circulating myeloid cells, and clinical ocular manifestations after chemical and mechanical injuries to the cornea. The injury was made by applying absolute ethanol to the cornea and scraping off its epithelium in BALB/c mice [28, 31]. At 1 min, 30 min, 2 h, 6 h, 1 d, 7 d, 14 d, 21 d and 28 d after injury, the corneas were observed clinically by slit-lamp biomicroscopy and harvested for immunostaining in order to detect corneal nerves, Ly6G^+^ cells and Ly6C^+^ cells (Fig. 1A). Also, peripheral blood cells were collected and subjected to flow cytometry for detection of CD11b, Ly6G and Ly6C expression.

The injury caused visually significant corneal stromal opacity and neovascularization as examined by slit-lamp biomicroscopy (Fig. 1A, B). Time-course analysis indicated that corneal epithelial defect was completely healed by 14 days after injury (day 14) in 80% of mice. Corneal opacity was most noticeable at day 7 and gradually decreased thereafter. Corneal neovascularization ensued thereafter, and reached a peak at days 21–28.

Corneal nerve density progressively declined with time after injury (Fig. 1C, D). The immunostaining of corneal whole-mounts with Ab against β-tubulin III (pan-neuronal marker) showed that both thick stromal nerves and thin sub-basal nerves were significantly lost, beginning within 1 min after injury and resulting in a maximal loss at days 1–7. Then, partial regeneration of deep stromal nerves followed but without recovery of the sub-basal hairpin-like nerve plexus until day 28 (Fig. 1C).

At the same time, the injury triggered a robust inflammatory response with an influx of myeloid cells into the cornea, of which Ly6G^+^ cells were the predominant population, covering up to 20% of the whole corneal area at day 7 as evaluated by Ly6G immunostaining of corneal flat-mounts (Fig. 1E, F). By comparison, there was less infiltration of Ly6C^+^ cells into the cornea, covering up to 5% of the corneal area at day 7 (Fig. 1G). Interestingly, the kinetics of Ly6G^+^ cells infiltration into the cornea were in parallel with those of corneal nerve loss. Ly6G^+^ cells, after injury, infiltrated the cornea progressively from the periphery to the center, reaching a peak at day 7 and disappearing over days 7 to 28 (Fig. 1E, F). A similar time course was observed for the percentage of CD11b^hi^Ly6G^hi^ cells in the peripheral blood, as analyzed by flow cytometry, whereas there was no change in the percentage of circulating CD11b^hi^Ly6C^hi^Ly6G^lo^ cells at day 1 or 7 (Fig. 1H, I).

The finding that the corneal infiltration of Ly6G^+^ cells corresponded to the reduction of corneal nerves led us to posit that Ly6G^+^ myeloid cells might play a role in corneal nerve loss after sterile injury. Alternatively, the finding that the number of corneal Ly6G^+^ cells peaked at day 7 (a time-point wherein corneal nerves started to recover) suggests a beneficial role of Ly6G^+^ cells in corneal nerve regeneration.

### CD11b^hi^Ly6G^hi^ cell depletion exacerbates corneal nerve loss while increasing CD11b^hi^Ly6C^hi^Ly6G^lo^ cells and IL-6

**Fig. 2:**
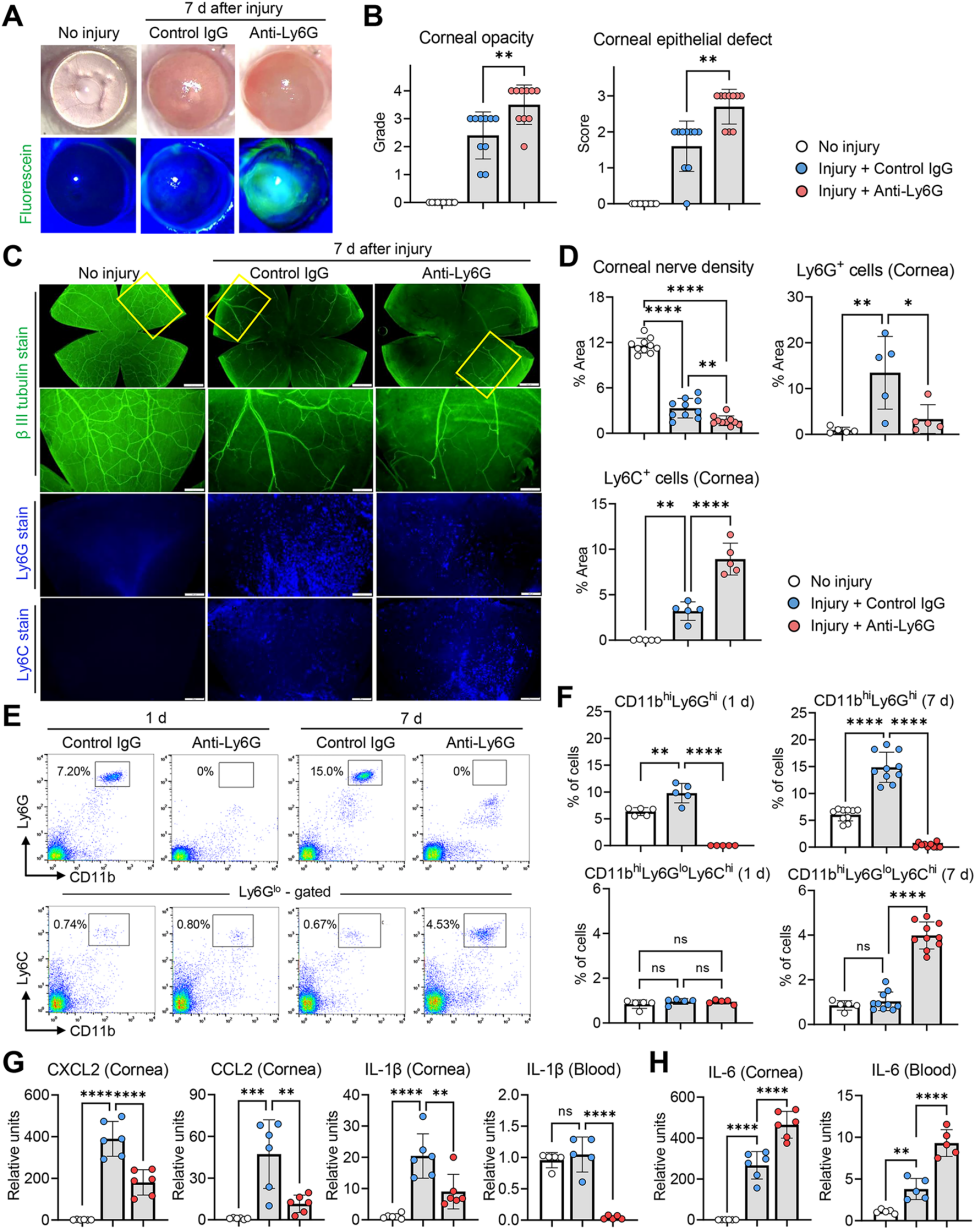
CD11b^hi^Ly6G^hi^ cell depletion aggravates corneal nerve loss while increasing CD11b^hi^Ly6C^hi^ cells and IL-6. **A**, **B** Representative corneal photographs with fluorescein staining at 7 d post-injury (**A**). The green-stained areas are those with epithelial damage. Quantitative analysis of corneal opacity and epithelial defect at 7 d post-injury (**B**). **C**, **D** Representative images of corneal flat-mounts with β-tubulin III, Ly6G and Ly6C staining at 7 d post-injury and higher magnification views of corneal quadrant in each cornea (yellow box) (**C**). Quantitative analysis of β-tubulin III-stained, Ly6G-stained or Ly6C-stained areas (**D**). **E**, **F** Flow cytometric analysis for CD11b^hi^Ly6G^hi^ cells and CD11b^hi^Ly6C^hi^Ly6G^lo^ cells in peripheral blood at 1 and 7 d post-injury. **G**, **H** Levels of pro-inflammatory chemokine/cytokine mRNAs in cornea and blood cells at 1 d post-injury as analyzed by real-time RT-PCR. The values are shown relative to those in healthy, naive mice without injury. Mean values ± SD are shown, where each circle depicts the data from an individual mouse. A white circle depicts the data from a healthy, naive mouse without injury. A blue circle depicts the data from an injured mouse treated with isotype control IgG (2A3) and a red circle from an injured mouse treated with anti-Ly6G Ab (1A8). *p < 0.05, **p < 0.01, ***p < 0.001, ****p < 0.0001, ns: not significant, as analyzed by one-way ANOVA and Tukey’s test, Mann–Whitney U test (corneal epithelial defect in **B**) or by Kruskal–Wallis test and Dunn’s multiple-comparison test (CD11b^hi^Ly6G^lo^Ly6C^hi^ (1 d) in **F**)

To investigate the role of Ly6G^+^ myeloid cells, we depleted CD11b^hi^Ly6G^hi^ cells in mice by IP injection of 1A8 Ab or isotype-matched control IgG after injury (Fig. 2A–H) [32, 33]. A significant 75% reduction in corneal Ly6G^+^ cells was noted by the immunohistochemistry of the corneal whole-mounts (Fig. 2C, D), and a near-complete depletion of systemic CD11b^hi^Ly6G^hi^ cells in the blood was confirmed by flow cytometry at both days 1 and 7 after injury (Fig. 2E, F).

Depletion of CD11b^hi^Ly6G^hi^ cells aggravated corneal stromal opacity, delayed corneal epithelial healing, and worsened corneal nerve loss after injury (Fig. 2A–D). Also, CD11b^hi^Ly6G^hi^ cell depletion increased the infiltration of Ly6C^+^ cells into the cornea (Fig. 2C, D). Similarly, the percentage of CD11b^hi^Ly6C^hi^Ly6G^lo^ cells in the blood was significantly increased in 1A8-treated mice at day 7 (4.0 ± 0.6% in 1A8-treated vs. 1.0 ± 0.4% in control IgG-treated mice, *p* < 0.0001) (Fig. 2E, F). The transcript levels of IL-1β, CCL2 and CXCL2 in the cornea or the blood at day 1 were significantly lower in mice treated with 1A8 Ab than in those treated with control IgG (Fig. 2G), which suggests that CD11b^hi^Ly6G^hi^ cells were the major source of these cytokines/chemokines. We chose to evaluate IL-1β, CCL2 and CXCL2 because they were the pro-inflammatory cytokines/chemokines that were gradually upregulated over 24 h post-injury in parallel with neutrophil infiltration as shown in our previous study [31]. By contrast, the levels of IL-6, one of the most highly upregulated cytokines upon corneal injury (up to 348-fold increase relative to normal cornea at day 1), were markedly upregulated in the cornea and the blood of 1A8-treated mice, as compared to control IgG-treated mice (Fig. 2H).

These results indicate that CD11b^hi^Ly6G^hi^ cells play a protective role in corneal nerve loss under sterile injury, notwithstanding the fact that they induce inflammation through the production of pro-inflammatory cytokines/chemokines. Remarkably, CD11b^hi^Ly6G^hi^ cell depletion led to reciprocal increases in CD11b^hi^Ly6C^hi^Ly6G^lo^ cells and IL-6 in both cornea and blood.

### CD11b^hi^Ly6C^hi^Ly6G^lo^ cell depletion aggravates corneal nerve loss while increasing IL-6

**Fig. 3:**
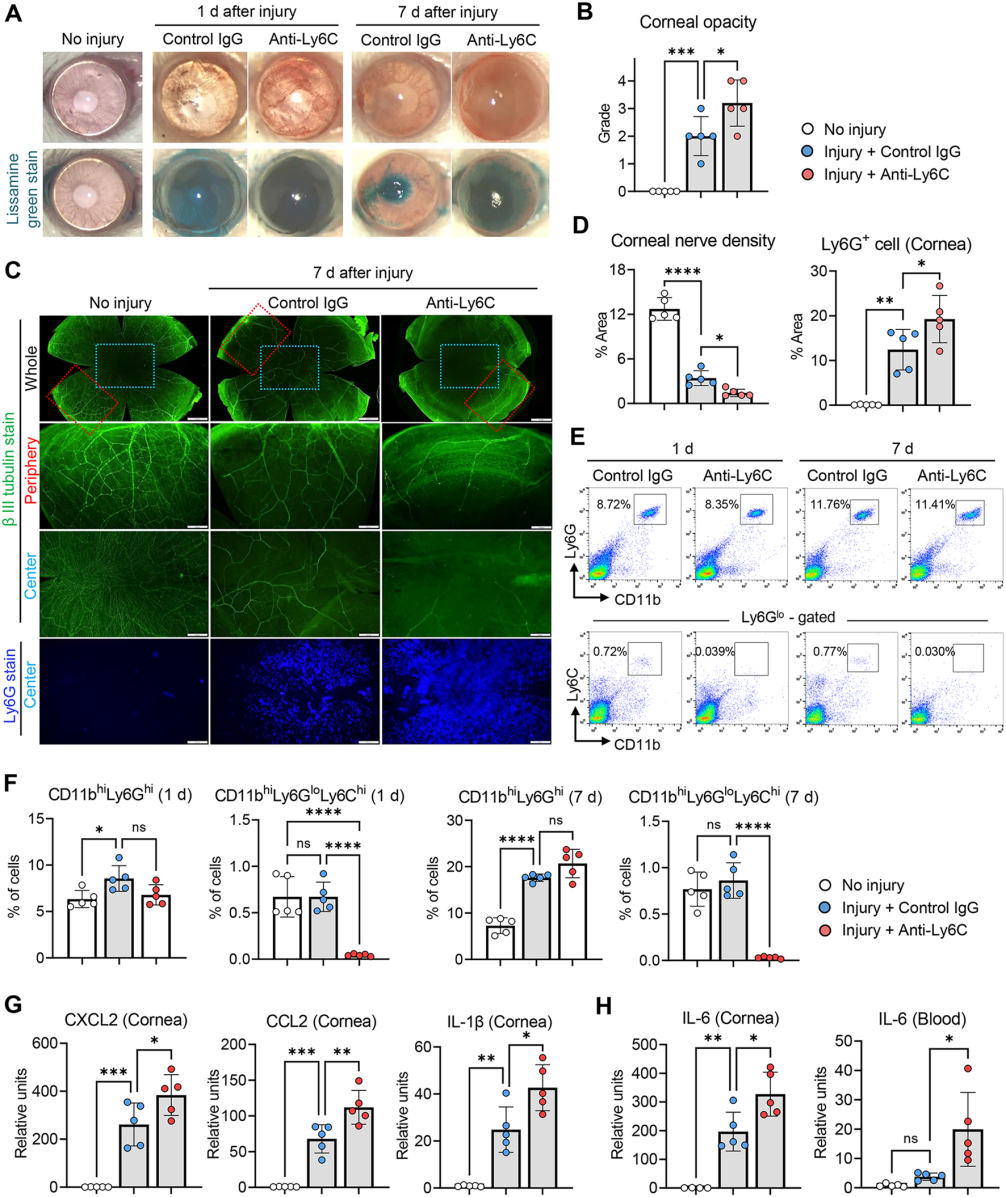
CD11b^hi^Ly6C^hi^ cell depletion augments corneal nerve loss while increasing IL-6. **A**, **B** Representative photographs of corneas before and after lissamine green vital dye staining (**A**). The green-stained areas indicate corneal epithelial defect. Quantification of corneal opacity at 7 d post-injury as graded by the standardized scoring system (0–4) (**B**). **C**, **D** Representative β-tubulin III and Ly6G staining images of corneal whole-mounts, with corneal center (blue box) and periphery oriented to limbus at top (red box) at 7 d post-injury (**C**). Analysis of corneal innervation and Ly6G^+^ cell infiltrates at 7 d post-injury quantified as percentage threshold area positive for β-tubulin III and Ly6G, respectively (**D**). **E**, **F** Representative flow cytometry cytograms (**E**) and quantitation of CD11b^hi^Ly6G^hi^ cells and CD11b^hi^Ly6C^hi^Ly6G^lo^ cells in peripheral blood at 1 and 7 d post-injury (**F**). **G**, **H** Real-time RT-PCR for pro-inflammatory chemokine/cytokine mRNAs in cornea and blood cells at 1 d post-injury. The mRNA levels are presented as fold changes relative to the levels in normal corneas or blood cells without injury. Mean values ± SD are presented, where each circle represents the data from an individual mouse. A white circle depicts the data from an uninjured, naive mouse, a blue circle from an injured mouse treated with isotype control IgG (2A3), and a red circle from an injured mouse treated with anti-Ly6C Ab (Monts-1). *p < 0.05, **p < 0.01, ***p < 0.001, ****p < 0.0001, ns: not significant, as analyzed by one-way ANOVA and Tukey’s multiple-comparison test

In the above experiments, after CD11b^hi^Ly6G^hi^ cell depletion, CD11b^hi^Ly6C^hi^Ly6G^lo^ cells were increased and corneal nerve loss got more severe (Fig. 2). We thus went on to evaluate whether CD11b^hi^Ly6C^hi^Ly6G^lo^ cells might contribute to corneal nerve damage after injury. To this end, we used IP injection of Monts-1 Ab in BALB/c mice for selective depletion of CD11b^hi^Ly6C^hi^Ly6G^lo^ cells (Fig. 3A–H) [34]. Near-complete depletion of CD11b^hi^Ly6C^hi^Ly6G^lo^ cells was confirmed by flow cytometry at both days 1 and 7 after injury (Fig. 3E, F). However, CD11b^hi^Ly6C^hi^Ly6G^lo^ cell depletion by Monts-1 Ab did not affect the percentage of CD11b^hi^Ly6G^hi^ cells in the blood (Fig. 3E, F).

Similarly to CD11b^hi^Ly6G^hi^ cell depletion (Fig. 2), depletion of CD11b^hi^Ly6C^hi^Ly6G^lo^ cells further aggravated corneal nerve loss and corneal opacity after injury (Fig. 3A–D), which indicates a protective role of CD11b^hi^Ly6C^hi^Ly6G^lo^ cells in corneal nerves against sterile injury. In contrast to CD11b^hi^Ly6G^hi^ cell depletion, the mRNA levels of IL-1β, CCL2 and CXCL2 in the cornea as well as corneal Ly6G^+^ cell infiltration were higher in mice treated with Monts-1 Ab, as compared to those treated with control IgG (Fig. 3C, D, G). Despite their opposite effects on IL-1β, CCL2 and CXCL2 levels, both CD11b^hi^Ly6G^hi^ and CD11b^hi^Ly6C^hi^Ly6G^lo^ cell depletion led to further loss of corneal nerves (Fig. 2D, 3D), and therefore, these cytokines were not implicated in corneal nerve loss. Importantly, however, the levels of IL-6 in the cornea and the blood were significantly enhanced in Monts-1-treated, CD11b^hi^Ly6C^hi^Ly6G^lo^ cell-depleted mice (Fig. 3H), a common finding in cases of CD11b^hi^Ly6G^hi^ cell depletion.

### IL-6 blockade attenuates, while IL-6 supplementation aggravates, corneal nerve loss

Based on the observation that both CD11b^hi^Ly6G^hi^ cell depletion (Fig. 2) and CD11b^hi^Ly6C^hi^Ly6G^lo^ cell depletion (Fig. 3) commonly led to upregulation of IL-6 alongside aggravation of corneal nerve loss, we next tested the possibility that IL-6 might be responsible for corneal nerve damage after sterile corneal injury. For this purpose, we locally blocked or replenished IL-6 in BALB/c mice by subconjunctival injection of neutralizing IL-6 Ab or recombinant IL-6 protein immediately after injury.

**Fig. 4:**
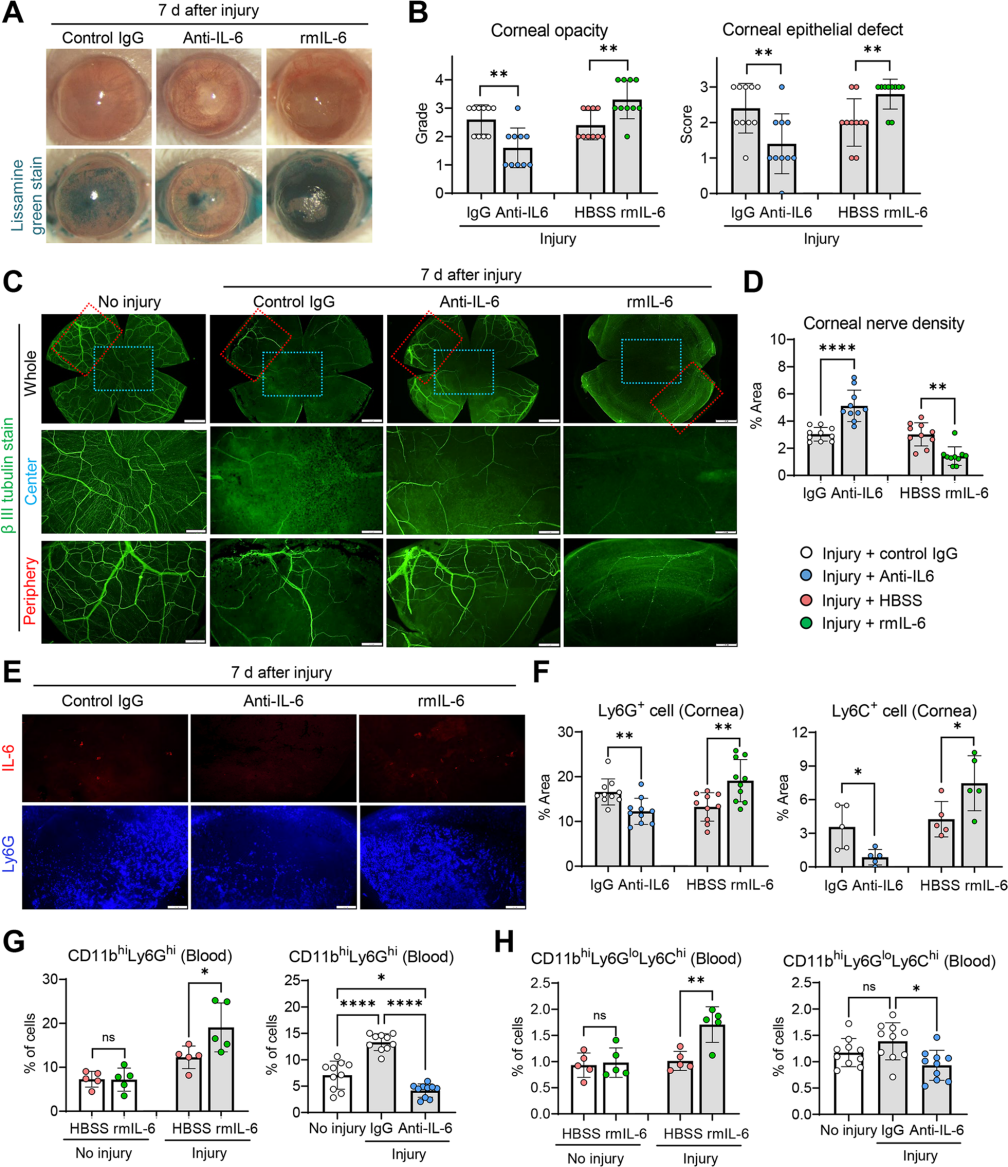
IL-6 neutralization prevents corneal nerve damage and IL-6 replenishment exacerbates it. **A**, **B** Representative corneal photographs without and with lissamine green staining for visualization of epithelial defect at 7 d post-injury (**A**). Severity of corneal opacity and epithelial defect as graded by standardized scoring systems (0–4 for corneal opacity and 0–3 for epithelial defect) (**B**). **C**–**F** Representative images of corneal whole-mounts, center (blue box) and periphery (red box) after β-tubulin III (**C**), IL-6 or Ly6G staining (**E**). The tissues were extracted at 7 d post-injury. Quantitative analysis of corneal nerve density (**D**) and corneal infiltration of Ly6G^+^ cells and Ly6C^+^ cells (**F**). **G**, **H** Flow cytometric analysis for CD11b^hi^Ly6G^hi^ cells and CD11b^hi^Ly6C^hi^Ly6G^lo^ cells in blood at 7 d post-injury. Mean values ± SD are shown, and each circle depicts the data from an individual mouse. A white circle indicates the data from control mice (either naive mice without injury or injured mice receiving isotype control IgG). A red circle indicates the data from another control mice receiving HBSS (Hank’s balanced salt solution, vehicle for recombinant mouse (rm) IL-6) after injury. A blue circle demonstrates the data from a mouse receiving anti-IL-6 Ab, and a green circle from a mouse receiving rmIL-6. *p < 0.05, **p < 0.01, ***p < 0.001, ****p < 0.0001, ns: not significant, as analyzed by one-way ANOVA and Tukey’s test or Mann–Whitney U test

The anti-IL-6 treatment significantly ameliorated clinical ocular manifestations after injury, as reflected by reduced corneal opacity and facilitated epithelial healing, whereas IL-6 addition deteriorated corneal opacity and epithelial defect (Fig. 4A, B). Corneal nerve density was significantly preserved in the anti-IL-6-treated mice, as compared to the control IgG-treated mice, while corneal nerves were further lost in mice treated with recombinant IL-6 (Fig. 4C, D). Moreover, the numbers of both cornea-infiltrating Ly6G^+^ cells and systemic CD11b^hi^Ly6G^hi^ cells were significantly repressed by anti-IL-6 treatment and, contrastingly, increased by IL-6 replenishment (Fig. 4E–G). Similar findings were observed with regard to the percentages of CD11b^hi^Ly6C^hi^Ly6G^lo^ cells in the cornea and the blood (Fig. 4F, H). It should be noted that neither anti-IL-6 nor recombinant IL-6 treatment had a significant impact on the cornea or systemic myeloid cells in the steady-state (i.e., in mice without injury) (Fig. 4G, H).

Collectively, the results indicate that IL-6, which is released at high levels in the cornea upon sterile injury, contributes to corneal nerve loss and induces recruitment of mostly Ly6G^+^ myeloid cells (and, to a lesser extent, Ly6C^+^ myeloid cells) to the cornea. The myeloid cells, in turn, protect corneal nerves by suppressing IL-6 through a negative-feedback loop.

### Absence of TLR2, not TLR4, reduces IL-6 and attenuates corneal nerve loss after injury

We further searched for the upstream signaling that stimulates IL-6 and induces corneal nerve loss upon sterile injury to the cornea. Given that activation of TLR2 elicits IL-6 trans-signaling [35], and also in light of our previous data that TLR2 signaling is the principal stimulus for innate immune response in the cornea after sterile injury [31], we determined to explore the roles of TLR2 and TLR4 by utilizing mice lacking TLR2 or TLR4.

**Fig. 5:**
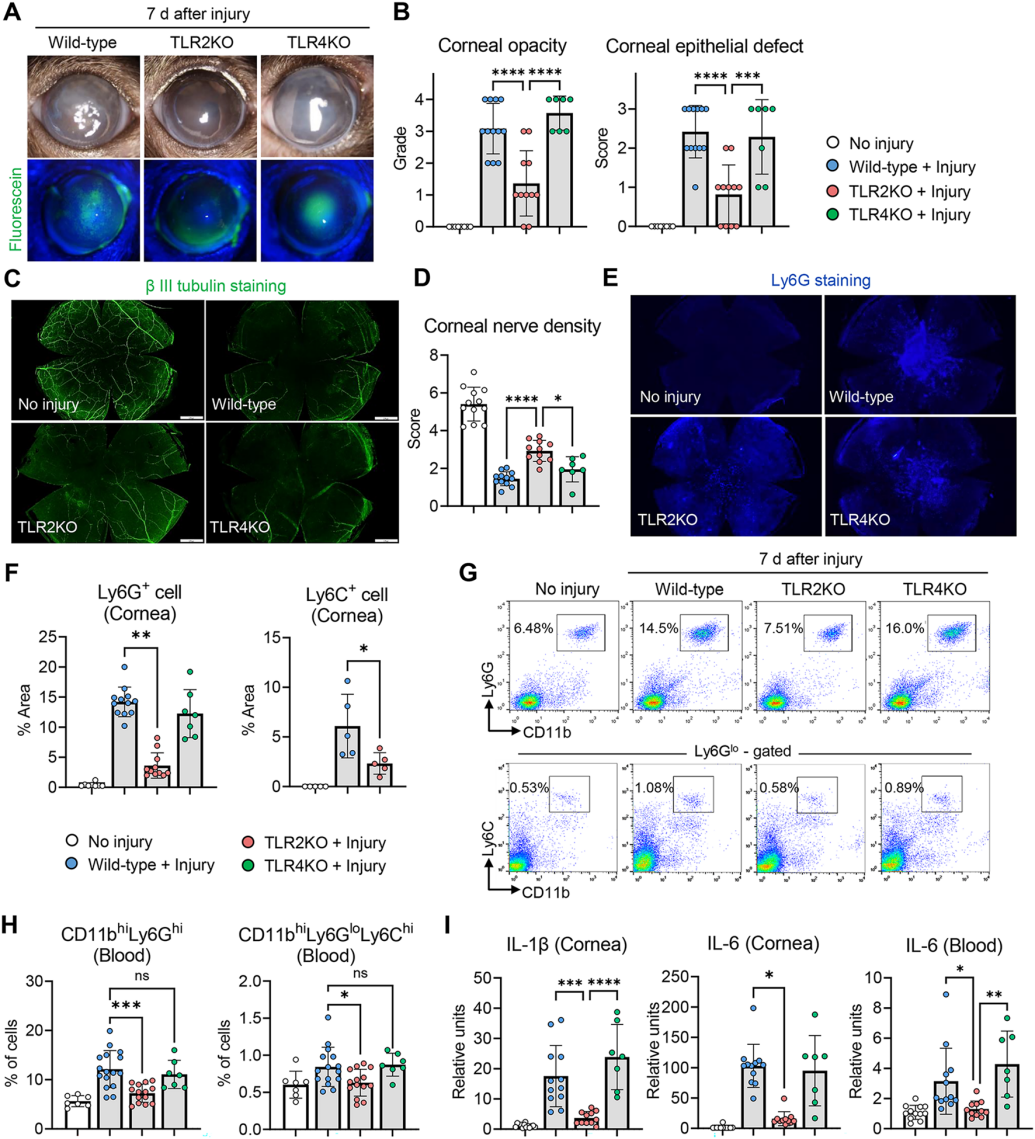
TLR2 KO mice exhibit attenuated corneal nerve loss alongside IL-6 downregulation after injury, compared to wild-type or TLR4 KO mice. **A**, **B** Representative corneal photographs with fluorescein staining in wild-type (C57BL/6J), TLR2 KO and TLR4 KO mice at 7 d post-injury (**A**). Quantitative analysis of corneal opacity and epithelial defect at 7 d post-injury (**B**). **C**–**F** Corneal whole-mount images stained with β-tubulin III (**C**) and Ly6G (**E**) at 7 d post-injury. Quantitative analysis of corneal innervation (**D**) and cornea-infiltrating Ly6G^+^ cells and Ly6C^+^ cells (**F**) at 7 d post-injury. **G**, **H** Representative flow cytometry cytograms (**G**) and quantitative analysis of CD11b^hi^Ly6G^hi^ cells and CD11b^hi^Ly6C^hi^Ly6G^lo^ cells in blood at 7 d post-injury (**H**). **I** mRNA levels of IL-1β and IL-6 in cornea and blood cells as measured by real-time RT-PCR. Values are shown relative to those in wild-type mice without injury. Mean values ± SD are presented, and each circle represents the data from an individual mouse. A white circle indicates the data from wild-type C57BL/6J mouse without injury, a blue circle from C57BL/6J mouse with injury, a red circle from TLR2 KO mouse with injury, and a green circle from TLR4 KO mouse with injury. *p < 0.05, **p < 0.01, ***p < 0.001, ****p < 0.0001, ns: not significant, as analyzed by one-way ANOVA and Tukey’s test or by Kruskal–Wallis test and Dunn’s multiple-comparison test (Ly6G^+^ cell in **F**, IL-6 (Cornea) in **I**)

Corneal nerve loss and clinical ocular manifestations (corneal stromal opacity and epithelial defect) were markedly attenuated in TLR2 KO mice after injury, as compared to wild-type C57BL/6 and TLR4 KO mice (Fig. 5A–D). Corneal infiltration of Ly6G^+^ cells and Ly6C^+^ cells as well as circulating CD11b^hi^Ly6G^hi^ cells and CD11b^hi^Ly6C^hi^Ly6G^lo^ cells in the blood were reduced in TLR2KO mice, as compared to wild-type or TLR4 KO mice (Fig. 5E–H). The IL-6 mRNA levels in the cornea and blood as well as the IL-1β level in the cornea were significantly lower in TLR2KO mice than in wild-type or TLR4 KO mice (Fig. 5I). On the other hand, no differences were observed in any examined parameters between TLR4 KO and wild-type mice. Therefore, the results demonstrate that TLR2, not TLR4, is a signaling receptor upstream of the IL-6-myeloid cell axis under sterile corneal injury.

### TLR2 stimulation induces IL-6 production in corneal stromal fibroblasts

We next sought to identify the cell population responsible for IL-6 production in response to TLR2 activation upon corneal injury. Since IL-6 was highly elevated in the cornea denuded of its epithelium at day 1 after injury (Figs. 2H, 3H, 5I), corneal epithelial cells are unlikely to be the source of IL-6. And because CD11b^hi^Ly6G^hi^ or CD11b^hi^Ly6C^hi^ cell depletion resulted in IL-6 increase (Figs. 2H, 3H), it is also unlikely that cornea-infiltrating Ly6G^+^ or Ly6C^+^ cells are the main IL-6 producers. We thus entertained the possibility that IL-6 might be secreted by keratocytes, which are fibroblasts and a major cell population in the corneal stroma representing 90% of the corneal thickness [36].

**Fig. 6:**
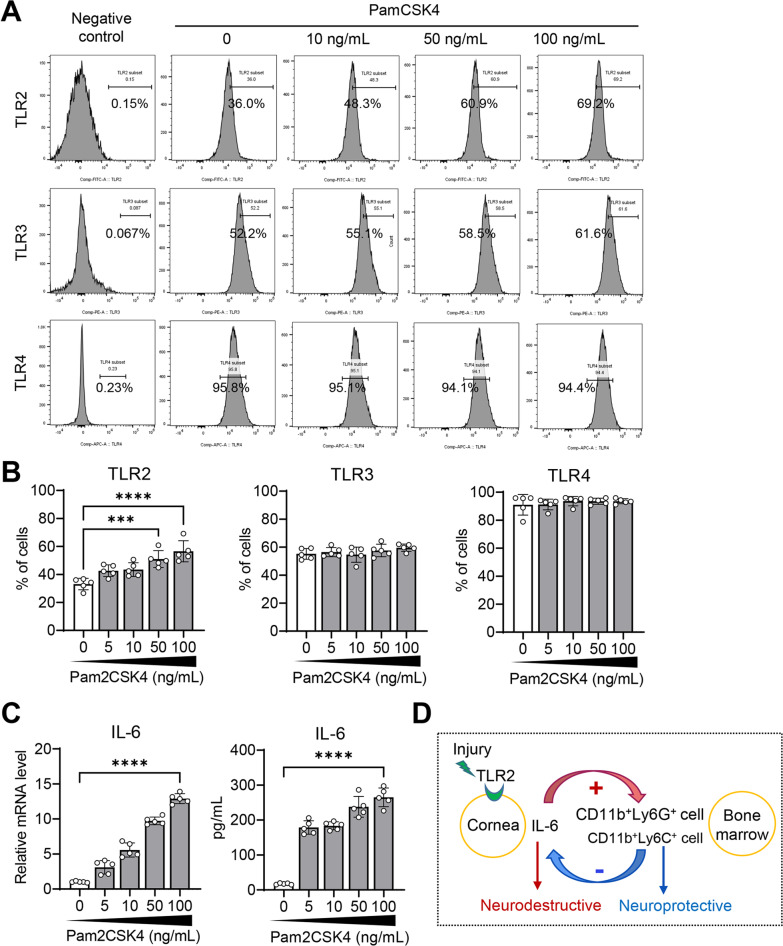
Corneal stromal fibroblasts are activated to produce IL-6 by TLR2 stimulation. **A**, **B** Representative flow cytometry cytograms (**A**) and quantitative analysis of TLR2, TLR3 and TLR4 in human keratocytes with or without 0–100 ng/mL of Pam2CSK4 treatment for 24 h. **C** Real-time RT-PCR for IL-6 in human keratocytes after Pam2CSK4 treatment and ELISA for IL-6 in the supernatant of primary human keratocytes. The mRNA levels are presented as fold changes relative to the levels in cells without Pam2CSK4 stimulation. **D** Schematic of proposed signaling pathway through which sterile injury leads to corneal nerve damage and homeostatic regulation limits corneal nerve loss. The data are presented in mean values ± SD, where each circle depicts data from an individual biological sample. ***p < 0.001, ****p < 0.0001, as analyzed by one-way ANOVA and Tukey’s multiple-comparison test

Primary cultures of keratocytes isolated from human corneal stroma were treated with various concentrations of Pam2csk4, a TLR2 agonist (0–100 ng/mL) for 24 h. Keratocytes expressed TLR2, TLR3 and TLR4 as assessed by flow cytometry. Pam2csk4 increased the expression levels of TLR2 in keratocytes in a dose-dependent manner, while TLR3 or TLR4 levels were not affected by Pam2csk4 (Fig. 6A, B). Also, Pam2csk4 upregulated the mRNA level of IL-6 and elicited the production of IL-6 protein in keratocytes in a dose-dependent manner (Fig. 6C). Hence, the data suggest that keratocytes express TLR2 and are activated to produce IL-6 by TLR2 stimulation.

## Discussion

In the present study, we identified the TLR2–IL-6 axis as the principal pathway driving corneal nerve loss upon sterile injury to the cornea. Activation of TLR2–IL-6 signaling induced recruitment of a large number of CD11b^hi^Ly6G^hi^ cells and, to a lesser extent, CD11b^hi^Ly6C^hi^Ly6G^lo^ cells. Both myeloid cell populations ameliorated the loss of corneal nerves through negative-feedback suppression of the upstream TLR2–IL-6 pathway (Fig. 6D). These results reveal one of the homeostatic mechanisms for regulation of corneal nerve damage under sterile injury, which mechanism, significantly, can be harnessed to uncover new therapeutic targets.

IL-6 is a pleiotropic cytokine critical for pathogen defense, the pathogenesis of inflammatory disorders, and tissue regeneration [37]. IL-6 is also involved in neurodegeneration and recovery, specifically by directly affecting neuronal survival and function or indirectly through neuroinflammatory pathways [38]. Studies have demonstrated that IL-6 is increased in the nervous system upon injury and mediates disease progression in peripheral neuropathies [38–43] as well as in neurodegenerative diseases of the CNS where neuroinflammation plays a role such as multiple sclerosis (MS), Parkinson's disease (PD) and Alzheimer's disease (AD) [38, 44–49]. Similarly, in the cornea, IL-6 is associated with corneal nerve dysfunction. Tear IL-6 level was shown to be inversely correlated with corneal nerve density in patients with bacterial keratitis [50]. The circulating level of IL-6 was significantly associated with corneal nerve fiber loss in patients with transient ischemic attack and acute ischemic stroke [51]. Moreover, an elegant study by Chucair-Elliott et al. [52] reported that IL-6 was increased in the murine cornea after herpes simplex virus (HSV) infection, and that IL-6 suppression by either neutralizing Ab or dexamethasone reduced corneal nerve regression, thereby preserving sensation in HSV keratitis. This notion that IL-6 contributes to corneal nerve degeneration is consistent with our findings. Furthermore, our study suggested that IL-6 can be produced by corneal cells involving keratocytes via TLR2 activation upon sterile corneal injury. In addition, IL-6 acted as a primary chemoattractant that induced recruitment of CD11b^hi^Ly6G^hi^ and CD11b^hi^Ly6C^hi^Ly6G^lo^ myeloid cells to the site of injury; eventually, the recruited myeloid cells negatively regulated IL-6 production and alleviated corneal nerve loss.

Infiltration of inflammatory leukocytes, including myeloid cells, into the nervous system is generally considered detrimental in the context of neuroinflammation and neurodegeneration. There is, however, accumulating evidence that myeloid cell subsets, such as alternatively activated M2 macrophages, N2 neutrophils and myeloid-derived suppressor cells (MDSCs), play an indispensable role in protecting nerves and improving neurological outcomes in multiple diseases of the brain [4, 6, 11, 53, 54], spinal cord [2, 8, 12] and optic nerve [2, 55]. In particular, neutrophils, the most abundant circulating myeloid cells and a major cell infiltrate in injured tissues, have recently emerged as important cell types conferring neuroprotective and regenerative effects [2, 4, 8, 53, 54, 56]. In our study, CD11b^hi^Ly6G^hi^ cells were highly increased in the blood and infiltrated into the cornea following injury, as compared to CD11b^hi^Ly6C^hi^Ly6G^lo^ cells, and the depletion of CD11b^hi^Ly6G^hi^ cells aggravated corneal nerve loss, demonstrating a critical role of the cells in corneal nerve protection against sterile injury. Consistent with our findings, other studies have demonstrated that N2 neutrophils expressing alternative activation markers, such as Arg1, YM1 or CD206 inhibited cortical neuron damage and promoted brain remodeling after ischemic injury [4, 53, 54, 56]. Also, a recent study by Sas et al. [2] elegantly showed that the CD11b^hi^Ly6G^lo^ neutrophil subset drove neuronal survival and axonal regeneration in traumatic optic nerve and spinal cord injury partly via secretion of a cocktail of neuroprotective growth factors. Transcriptomic analysis by single-cell RNA sequencing, in that study, revealed that the neuroregenerative Ly6G^lo^ neutrophils fell within the immature neutrophil cluster and exhibited alternative activation markers Arg1, CD206 and IL4ra. Given the rapidly evolving understanding of neutrophil heterogeneity [57], further study is needed to phenotypically and functionally characterize CD11b^hi^Ly6G^hi^ cells, which, we found, were systemically mobilized, were recruited to the cornea, and conferred neuroprotection after corneal injury.

In our study and setting, TLR2 signaling was responsible for IL-6 production and subsequent myeloid cell recruitment in the cornea under sterile injury, whereas TLR4 did not affect either corneal pathology or systemic myeloid cell mobilization. This finding is in agreement with our previous observation that TLR2/NF-κB signaling was a crucial pattern recognition receptor that recognized a damage/danger-associated molecular pattern HSPB4 generated by the cornea upon sterile injury and triggered the release of a cascade of pro-inflammatory cytokines [31]. TLR2 is expressed by many cell types such as innate immune cells, resident corneal cells and neurons. Activation of TLR2 signaling initiates a pro-inflammatory response and thereby drives the pathogenesis of neurodegeneration in a wide array of disorders ranging from infection-induced neuroinflammation to sterile neuroinflammation-associated conditions including stroke, MS, AD, PD and neuropathic pain [58, 59]. In the present study, we showed that keratocytes (corneal stromal fibroblasts) were capable of IL-6 secretion in response to TLR2 stimulation in culture, raising the possibility that keratocytes are one of the sources of IL-6 release after sterile corneal injury. However, it is also possible that IL-6 is released from resident macrophages or corneal nerves, because these cell types also express TLR2 and are capable of producing its downstream effector IL-6 [58]. Further study would be needed to identify the main cell type responsible for IL-6 secretion in the cornea after injury.

## Conclusions

Homeostatic regulation among various players in disease pathogenesis is critical for tissue protection and regeneration upon injury. Our study identified a TLR2–IL-6–myeloid cell pathway that induces loss of corneal nerves after injury and subsequently activates negative-feedback regulation for their protection. Understanding this homeostatic mechanism that limits excessive corneal nerve loss would help open up new avenues for therapeutic targets and intervention in neuroinflammatory diseases of the PNS including the cornea.

## Data Availability

All data generated or analyzed during this study are included in this published article.

## Abbreviations

AD: Alzheimer's disease
CNS: Central nervous system
HSV: Herpes simplex virus
IP: Intraperitoneal
KO: Knockout
MDSC: Myeloid-derived suppressor cell
MS: Multiple sclerosis
PBS: Phosphate-buffered solution
PD: Parkinson's disease
PNS: Peripheral nervous system
RT: Room temperature
TLR: Toll-like receptor
